# *Notes from the Field:* Outbreak of Severe Illness Linked to the Vitamin K Antagonist Brodifacoum and Use of Synthetic Cannabinoids — Illinois, March–April 2018

**DOI:** 10.15585/mmwr.mm6721a4

**Published:** 2018-06-01

**Authors:** Erin Moritz, Connie Austin, Michael Wahl, Carol DesLauriers, Livia Navon, Kelly Walblay, Monica Hendrickson, Angie Phillips, Janna Kerins, Audrey F. Pennington, Amy M. Lavery, Tharwat El Zahran, Judy Kauerauf, Luke Yip, Jerry Thomas, Jennifer Layden

**Affiliations:** ^1^Epidemic Intelligence Service, CDC; ^2^Communicable Disease Control Section, Illinois Department of Public Health; ^3^Illinois Poison Center, Illinois Health and Hospital Association, Chicago, Illinois; ^4^Division of State and Local Readiness, Office of Public Health Preparedness and Response, CDC; ^5^Council of State and Territorial Epidemiologists Applied Epidemiology Fellowship; ^6^Peoria City/County Health Department, Illinois; ^7^Tazewell County Health Department, Tremont, Illinois; ^8^City of Chicago Department of Public Health, Illinois; ^9^Division of Environmental Health Science and Practice, National Center for Environmental Health, CDC; ^10^Geospatial Research Analysis and Services Program, National Center for Environmental Health/Agency for Toxic Substances and Disease Registry, CDC; ^11^Emory University School of Medicine, Atlanta, Georgia; ^12^Division of Laboratory Sciences, National Center for Environmental Health, CDC.

Synthetic cannabinoids, also known as K2 and spice, are heterogeneous psychoactive compounds identified as substances of abuse (*1*,*2*). On March 22, 2018, the Illinois Department of Public Health was notified by the Illinois Poison Center of four patients seen in emergency departments (EDs) during the preceding 2 weeks with unexplained bleeding and high international normalized ratios (INRs; range from 5 to >20 [normal <1.1]), indicating a clotting disorder, and reported synthetic cannabinoid use during the previous 3 days. None reported taking prescription anticoagulants or exposure to anticoagulant rodenticides. An investigation by the Illinois Department of Public Health, the Illinois Poison Center, CDC, local health departments, and law enforcement agencies was initiated to identify additional cases, ascertain epidemiologic links among patients, and implement control measures.

Requests for information regarding patients with serious bleeding and an elevated INR without a definitive etiology identified on or after February 1, 2018, were issued to Illinois EDs, emergency medical services, health care providers, local health departments, and coroners through Epi-X,* state and local health alert systems, and electronic distribution lists. Syndromic surveillance queries were developed by the Illinois Department of Public Health and implemented to identify patients evaluated at EDs and urgent care centers. Seven press releases encouraged anyone with a serious reaction after using synthetic cannabinoids to seek immediate medical attention.

Based on clinical signs and symptoms (unexplained bleeding, prolonged high INR values, and response to fresh frozen plasma and high doses of vitamin K), exposure to a long-acting vitamin K antagonist was suspected. Case definitions were developed based on signs and symptoms, synthetic cannabinoid exposure, and laboratory findings (Box). Data concerning signs and symptoms; synthetic cannabinoid use, brand, and location of purchase; and exposure to rodenticides, prescription anticoagulants, and illicit drugs were collected through patient interviews, medical chart abstraction, and Illinois Poison Center consultations. Blood samples were tested for presence of anticoagulants by high-performance liquid chromatography–tandem mass spectrometry (NMS Laboratories, Willow Grove, Pennsylvania).

BOX **Case definitions for unexplained bleeding after use of synthetic cannabinoids — Illinois, 2018**

**Clinical criteria**

Bruising, nosebleeds, bleeding gums, bleeding disproportionate to injury, vomiting blood, coughing up blood, blood in urine or stool, or excessively heavy menstrual bleeding.

**Laboratory criteria**

- Elevated international normalized ratios (INRs; ≥2.0) or abnormal coagulation profile (e.g., prothrombin time in absence of INR values) for which there is no other clinical explanation, or
- Detection of a long-acting anticoagulant (e.g., brodifacoum) in blood, serum, plasma, or urine, as determined by reference laboratory testing.

**Case classification**

**Suspected case**

One or more of the clinical criteria listed above in a patient, without an alternative explanation, and with reported use of synthetic cannabinoids or unknown drugs, or with some suspicion of previous or current drug use or exposure.

**Probable case**

- One or more of the clinical criteria listed above in a patient with reported use of synthetic cannabinoids in the 3 months preceding illness onset (by patient, proxy, medical record, or health care provider), and laboratory evidence of coagulopathy as measured by meeting the first laboratory criterion listed above, or
- One or more of the clinical criteria listed above, and meeting both laboratory criteria listed above, with no other explanation of results.

**Confirmed case**

One or more of the clinical criteria listed above in a patient, with reported use of synthetic cannabinoids in the 3 months preceding illness onset (by patient, proxy, medical record, or health care provider), and meeting the second laboratory criterion listed above.

As of April 25, 2018, a total of 155 cases (76 confirmed and 79 probable) had been identified; four (2.6%) patients died from major bleeding events. Median patient age was 32 years (range = 18–65 years), 115 (74%) were male, 81 (52%) were non-Hispanic white, 147 (95%) were hospitalized, and eight (5%) were treated in an ED only. The most frequently reported sign was hematuria (125; 81%); all patients reported bleeding from at least one site. INRs were elevated in all patients. All 81 (52%) analyzed clinical specimens from patients with a confirmed or probable case were positive for brodifacoum, a long-acting vitamin K antagonist used in rodenticides. Although cases clustered in two geographic areas (the Chicago area and seven neighboring counties in central Illinois), no single product source has been identified. Law enforcement is investigating the synthetic cannabinoid distribution network. Thirty-eight patients have been identified in eight other states, and CDC is conducting a multistate investigation (*3*). Product testing is ongoing in Illinois; some products in other states have tested positive for brodifacoum (*4*). Currently, the reason why brodifacoum was present in the synthetic cannabinoids is not known.

In 2017, 26 synthetic cannabinoids were listed as Schedule I substances under the Controlled Substances Act (*5*). However, they are often marketed as alternatives to marijuana or labeled as not for human consumption (*5*,*6*). They remain available for purchase, are relatively inexpensive, and are sometimes favored over marijuana because they are not detected in routine drug testing (*2*,*6*). The synthetic cannabinoid supply chain is unregulated, resulting in variable product compositions (*2*). Given the various compounds and unclear provenance, use of these products can result in unpredictable health effects (*1*). Stronger public messaging is needed and should target persons at risk. Engaging substance abuse services and community coalitions might improve outreach. Health care providers should consider vitamin K-dependent coagulopathy in patients with unexplained bleeding and reported or suspected synthetic cannabinoid use.

## Footnotes

* https://www.cdc.gov/mmwr/epix/epix.html.
